# Characterization of a novel Gram‐stain‐positive anaerobic coccus isolated from the female genital tract: Genome sequence and description of *Murdochiella vaginalis* sp. nov.

**DOI:** 10.1002/mbo3.570

**Published:** 2018-05-09

**Authors:** Khoudia Diop, Awa Diop, Saber Khelaifia, Catherine Robert, Fabrizio Di Pinto, Jérémy Delerce, Didier Raoult, Pierre‐Edouard Fournier, Florence Bretelle, Florence Fenollar

**Affiliations:** ^1^ Aix‐Marseille Univ Unité de Recherche sur les Maladies Infectieuses et Tropicales Emergentes UM 63 CNRS UMR 7278 IRD 198 INSERM U1095 Institut Hospitalo‐Universitaire Méditerranée‐Infection Faculté de Médecine Marseille France; ^2^ Special Infectious Agents Unit King Fahd Medical Research Center King Abdulaziz University Jeddah Saudi Arabia; ^3^ Department of Gynecology and Obstetrics Gynépole Marseille Hôpital Nord Assistance Publique‐Hôpitaux de Marseille Marseille France

**Keywords:** bacterial vaginosis, culturomics, genome, *Murdochiella vaginalis*, taxono‐genomics, vaginal microbiota

## Abstract

Strain Marseille‐P2341^T^, a nonmotile, nonspore‐forming, Gram‐stain‐positive anaerobic coccus, was isolated in the vaginal specimen of a patient with bacterial vaginosis using culturomics. Its growth occurred at temperatures ranging from 25 to 42°C, with pH between 6.5 and 8.5, and at NaCl concentrations lower than 5%. The major fatty acids were C_18:1n9_ (27.7%) and C_16:0_ (24.4%). Its genome is 1,671,491 bp long with 49.48 mol% of G+C content. It is composed of 1,501 genes: 1,446 were protein‐coding genes and 55 were RNAs. Strain Marseille‐P2341^T^ shared 97.3% of 16S rRNA gene sequence similarity with *Murdochiella asaccharolytica*, the phylogenetically closest species. These results enabled the classification of strain Marseille‐P2341^T^ as a new species of the genus *Murdochiella* for which we proposed the name *Murdochiella vaginalis* sp. nov. The type strain is strain Marseille‐P2341^T^ (=DSM 102237, =CSUR P2341).

## 1 INTRODUCTION

Due to vaginal secretions and, sometimes, urine, the vagina is a humid biotope which constitutes a complex ecosystem colonized by several types of microorganisms (Pal et al., 2011). Its composition was described for the first time in 1892 by Döderlein, who revealed that the vaginal flora is homogeneous and composed of Gram‐positive bacteria known as Döderlein bacilli (Lepargneur & Rousseau, 2002). Since then, many studies have been conducted, some of which suggest that this complex ecosystem is mostly dominated by the *Lactobacillus* genus (De Vos et al., 2009) with four main species: *Lactobacillus crispatus, Lactobacillus gasseri, Lactobacillus jensenii,* and *Lactobacillus vaginalis*. This constitutes the first line of defense against genital infections (Bohbot & Lepargneur, 2012; Turovskiy, Sutyak Noll, & Chikindas, 2011). An imbalance in this flora is observed in bacterial vaginosis.

The vaginal microflora diversity of a patient suffering from bacterial vaginosis was first described by Schröder in 1921 (Pal et al., 2011). This dysbiosis is characterized by a progressive decrease or even a lack of normal *Lactobacillus* flora accompanied by an increased pH of the vaginal lumen and an abnormal proliferation of previously underrepresented bacteria and Gram‐stain‐negative anaerobic bacteria (*Gardnerella vaginalis, Atopobium vaginae, Mobiluncus curtisii*, etc.) (Pépin et al., 2011; Shipitsyna et al., 2013). The mechanism of bacterial vaginosis is unknown; its empirical treatment and relapse rate is estimated at 50% at 3 months (Bretelle et al., 2015). This disturbance is associated with some complications in pregnant women such as miscarriage, chorioamnionitis, and preterm birth (Bretelle et al., 2015; Svare, Schmidt, Hansen, & Lose, 2006).

Initially studied using conventional culture methods, the understanding of the human vaginal microbiota was enhanced through the use of molecular techniques involving sequencing and phylogenetic analysis of the 16S rRNA gene (Lamont et al., 2011). These molecular methods enabled the detection of fastidious and uncultured bacteria such as bacterial vaginosis‐associated bacteria (BVAB): BVAB1 BVAB2, and BVAB3 (Fredricks, Fiedler, & Marrazzo, 2005). In order to identify all bacteria (uncultured and fastidious) present in the vagina and involved in this alteration, we studied normal vaginal flora and those from bacterial vaginosis using the concept of “microbial culturomics,” based on the multiplication of culture conditions with variations in temperature, media, pH, and atmospheric conditions, and rapid bacterial identification using matrix‐assisted laser‐desorption/ionization (MALDI) time‐of‐flight (TOF) mass spectrometry (MS) (Lagier et al., 2012, 2015). This microbial culturomics approach enabled us to isolate a new member of the *Murdochiella* genus that did not correspond to other species of this genus. This strain is designated as Marseille‐P2341^T^. The *Murdochiella* genus was created in 2010, to include strain recovered from a human abdominal wall abscess and in a sacral pilonidal cyst aspirate (Ulger‐Toprak, Liu, Summanen, & Finegold, 2010). This genus has only one valid species: *Murdochiella asaccharolytica*.

The description of new bacterial species is based on phenotypic and genotypic characteristics but has some limitations (Chan, Halachev, Loman, Constantinidou, & Pallen, 2012; Vandamme et al., 1996). In this manuscript we use taxonogenomics, a new approach combining classic characteristics with the proteomic information obtained from MALDI‐TOF MS and the description of the annotated whole genome (Fournier & Drancourt, 2015; Fournier, Lagier, Dubourg, & Raoult, 2015), to describe *Murdochiella vaginalis* sp. nov. (=DSM 102237 = CSUR P2341).

## 2 MATERIALS AND METHODS

### 2.1 Sample ethics and strain isolation

Using a Sigma Transwab (Medical Wire, Corsham, United Kingdom), the vaginal specimen of a 33‐year‐old French woman was collected and transported to the La Timone hospital in Marseille (France). Diagnosed as previously reported (Menard, Fenollar, Henry, Bretelle, & Raoult, 2008), the patient was suffering from bacterial vaginosis. At the time the sample was collected, she was not being treated with any antibiotics. The study was authorized by the local IFR48 ethics committee (Marseille, France) under agreement number 09‐022 and the patient also signed written consent. After sampling, the specimen was preincubated in a blood culture bottle (BD Diagnostics, Le Pont‐de‐Claix, France) enriched with 4 ml of rumen that was filter‐sterilized through a 0.2 μm pore filter (Thermo Fisher Scientific, Villebon‐sur‐Yvette, France) and 3 ml of sheep's blood (bioMérieux, Marcy l'Etoile, France). After different preincubation periods (1, 3, 7, 10,15, 20, and 30 days), 50 μl of the supernatant was inoculated on Schaedler agar (BD Diagnostics) and then incubated for 7 days under anaerobic conditions at 37°C.

### 2.2 Strain identification by MALDI‐TOF MS and 16S rRNA gene sequencing

Isolated colonies were deposited in duplicate on a MTP 96 MALDI‐TOF target plate (Bruker Daltonics, Leipzig, Germany) for identification with a microflex spectrometer (Bruker), as previously described (Seng et al., 2009). All obtained protein spectra were loaded into the MALDI Biotyper Software (Bruker Daltonics) and compared, as previously described (18), using the standard pattern‐matching algorithm, which compared the acquired spectrum with those present in the library (the Bruker database and our constantly updated database). If the score was greater than 1.9, the bacterium was considered to be identified at the species level. If not, identification failed and to achieve identification for unidentified colonies, the 16S rRNA gene was sequenced using fD1‐rP2 primers (Eurogentec, Angers, France) and the obtained sequence was matched against the NCBI database using the BLAST algorithm (Drancourt et al., 2000). As suggested, if the 16S rRNA gene sequence similarity value was lower than 95% or 98.7%, the strain was defined as a new genus or species, respectively (Kim, Oh, Park, & Chun, 2014; Stackebrandt & Ebers, 2006).

### 2.3 Phylogenetic analysis

All species from the same order of the new species were retrieved and 16S sequences were download from NCBI, by parsing NCBI eUtils results and the NCBI taxonomy page. Sequences were aligned using CLUSTALW, with default parameters and phylogenetic inferences obtained using the neighbor‐joining method with 500 bootstrap replicates, within MEGA6 software.

### 2.4 Growth conditions and morphological observation

To evaluate ideal growth, the strain Marseille‐P2341^T^ was cultivated on Columbia agar with 5% sheep's blood (bioMérieux) and incubated at different temperatures (25, 28, 37, 45, and 56°C) in an aerobic atmosphere with or without 5% CO_2_, and in anaerobic and microaerophilic atmospheres, using GENbag Anaer and GENbag microaer systems (bioMérieux). The salinity and pH conditions were also tested at different concentrations of NaCl (0%, 5%, 15%, and 45%) and different pH (5, 6, 6.5, 7, and 8.5).

Oxidase and catalase tests, Gram‐stain, motility, and sporulation were performed using standard procedures (Murray, Baron, Jorgensen, Landry, & Pfaller, 2007). To observe cell morphology, they were fixed with 2.5% glutaraldehyde in 0.1 mol/L cacodylate buffer for at least 1 hr at 4°C. A drop of cell suspension was then deposited for approximately 5 min on glow‐discharged formvar carbon film on 400 mesh nickel grids (FCF400‐Ni, EMS). The grids were dried on blotting paper and cells were negatively stained for 10 s with 1% ammonium molybdate solution in filtered water at RT. Electron micrographs were acquired using a Tecnai G20 Cryo (FEI) transmission electron microscope operated at 200 keV.

### 2.5 Biochemical and antibiotic susceptibility tests

Biochemical tests were performed using API ZYM, API 20A, and API 50CH strips (bioMérieux) according to the manufacturer's instructions. The strips were incubated for 4, 24, and 48 hr respectively.

Cellular fatty acid methyl ester (FAME) analysis was performed using Gas Chromatography/Mass Spectrometry (GC/MS). Strain Marseille‐P2341^T^ was grown on Columbia agar enriched with 5% sheep's blood (bioMérieux). Two samples were then prepared with approximately 50 mg of bacterial biomass per tube harvested from several culture plates. Fatty acid methyl esters were prepared as described by Sasser (Sasser, 2006). GC/MS analyses were carried out as previously described (Dione et al., 2016). In brief, fatty acid methyl esters were separated using an Elite 5‐MS column and monitored by mass spectrometry (Clarus 500—SQ 8 S, Perkin Elmer, Courtaboeuf, France). A spectral database search was performed using MS Search 2.0 operated with the Standard Reference Database 1A (NIST, Gaithersburg, USA) and the FAMEs mass spectral database (Wiley, Chichester, UK).

Antibiotic susceptibility was tested using the disc diffusion method (Le Page et al., 2015). The results were read using Scan 1200 (Interscience, Saint‐Nom‐la‐Bretèche, France).

### 2.6 Genomic DNA preparation

Genomic DNA (gDNA) of strain Marseille‐P2341^T^ was extracted in two steps: a mechanical treatment was first performed using acid‐washed glass beads (G4649‐500 g Sigma) and a FastPrep BIO 101 instrument (Qbiogene, Strasbourg, France) at maximum speed (6.5) for 3 × 30 s. Then after 2 hr of lysozyme incubation at 37°C, DNA was extracted on the EZ1 biorobot (Qiagen, Hilden, Germany) using the EZ1 DNA tissue kit. The elution volume was 50 μl. The gDNA was quantified by a Qubit assay using the high sensitivity kit (Life technologies, Carlsbad, CA, USA) to 103 ng/μl.

### 2.7 Genome sequencing and assembly

gDNA was sequenced on the MiSeq Technology (Illumina Inc, San Diego, CA, USA) using the mate pair strategy. The gDNA was barcoded using the Nextera Mate Pair sample prep kit (Illumina) to be mixed with 11 other projects. The mate pair library was prepared with 1.5 μg of genomic DNA using the Nextera mate pair Illumina guide. The genomic DNA sample was simultaneously fragmented and tagged with a mate pair junction adapter. The pattern of fragmentation was validated on an Agilent 2100 BioAnalyzer (Agilent Technologies Inc, Santa Clara, CA, USA) with a DNA 7500 labchip. The DNA fragments ranged in size from 1.5 kb to 11 kb with an optimal size at 3.716 kb. No size selection was performed and 652 ng of tagmented fragments were circularized. The circularized DNA was mechanically sheared to small fragments with a bi‐modal pattern at 644 bp and 1,613 bp on the Covaris device S2 in T6 tubes (Covaris, Woburn, MA, USA). The library profile was visualized on a High Sensitivity Bioanalyzer LabChip (Agilent Technologies Inc, Santa Clara, CA, USA) and the final concentration library was measured at 53.40 nmol/L.

The libraries were normalized at 2 nmol/L and pooled. After a denaturation step and dilution at 15 pmol/L, the pool of libraries was loaded onto the reagent cartridge and then onto the instrument along with the flow cell. Automated cluster generation and sequencing run were performed in a single 39‐hr run in a 2 × 251‐bp.

In total, 9.2 Gb of information was obtained from a 1042 K/mm^2^ cluster density with a cluster passing quality control filters of 91.6% (18,078,000 passing filter paired reads). Within this run, the index representation for strain Marseille‐P2341^T^ was determined to 13.14%. The 2,375,075 paired reads were trimmed then assembled in a scaffold.

### 2.8 Genome annotation and analysis

Prodigal was used for open reading frame (ORF) prediction (Hyatt et al., 2010) with default parameters. We excluded predicted ORFs spanning a sequencing gap region (containing *N*). The bacterial proteome was predicted using BLASTP (E‐value 1e^−03^ coverage 0.7 and identity percent 30) against the Clusters of Orthologous Groups (COG) database. If no hit was found, we searched against the NR database (Clark, Karsch‐Mizrachi, Lipman, Ostell, & Sayers, 2016), using BLASTP with E‐value of 1e^−03^ coverage 0.7 and an identity percent of 30. An E‐value of 1e^−05^ was used if sequence lengths were shorter than 80 amino acids. PFam conserved domains (PFAM‐A an PFAM‐B domains) were searched on each protein with the hhmscan tools analysis. RNAmmer (Lagesen et al., 2007) and tRNAScanSE tools (Lowe & Eddy, 1997) were used to find ribosomal RNAs genes and tRNA genes, respectively. ORFans were identified if all the BLASTP performed had negative results (E‐value smaller than 1e^−03^ for ORFs with sequence size above 80 aa or E‐value smaller than 1e^−05^ for ORFs with sequence length below 80 aa). For data management and visualization of genomic features, Artemis (Carver, Harris, Berriman, Parkhill, & McQuillan, 2012) and DNA Plotter (Carver, Thomson, Bleasby, Berriman, & Parkhill, 2009) were used, respectively. We used the home‐made MAGI software to analyze the mean level of nucleotide sequence similarity at the genome level. It calculated the average genomic identity of gene sequences (AGIOS) among compared genomes (Ramasamy et al., 2014). This software combines the Proteinrtho software (Lechner et al., 2011) for detecting orthologous proteins in pairwise genomic comparisons. The corresponding genes were then retrieved and the mean percentage of nucleotide sequence identity among orthologous ORFs was determined using the Needleman‐Wunsch global alignment algorithm. The Multi‐Agent software system DAGOBAH (Gouret et al., 2011) was used to perform the annotation and comparison processes, which included Figenix (Gouret et al., 2005) libraries for pipeline analysis. We also performed GGDC analysis using the GGDC web server, as previously reported (Meier‐Kolthoff, Auch, Klenk, & Göker, 2013).

## 3 RESULTS

### 3.1 Strain identification

**Figure 1:**
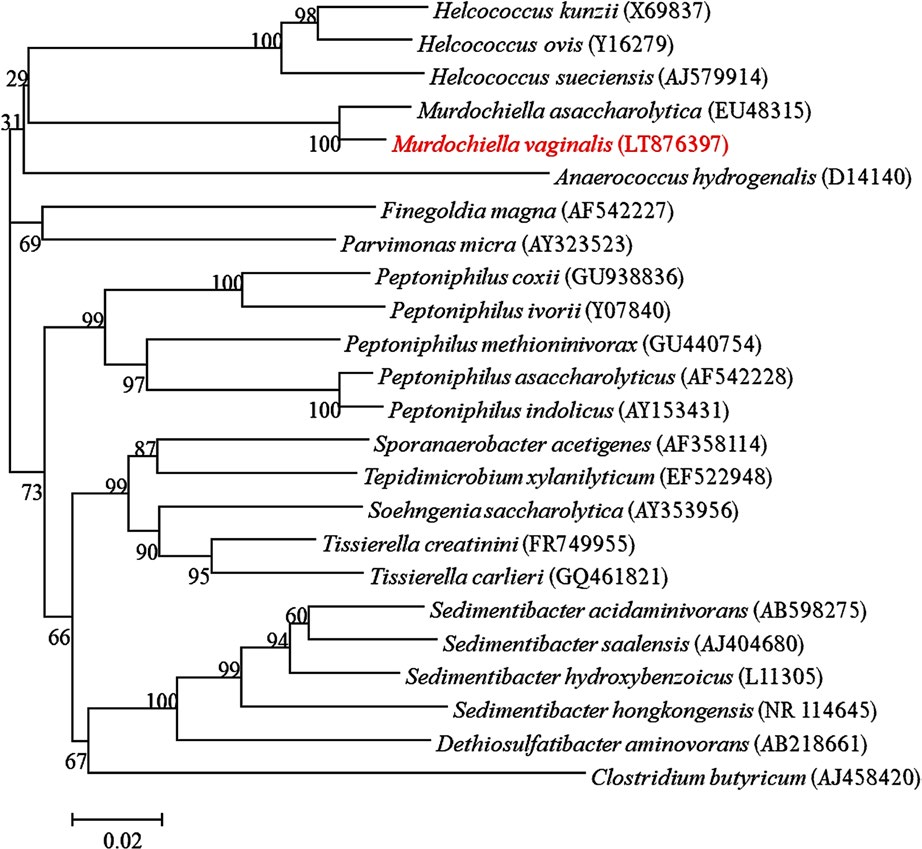
Phylogenetic tree highlighting the position of *Murdochiella vaginalis* strain Marseille‐P2341^T^ relative to other close strains. GenBank accession numbers of each 16S rRNA are noted in parenthesis. Sequences were aligned using Muscle v3.8.31 with default parameters and phylogenetic inferences were obtained using the neighbor‐joining method with 500 bootstrap replicates, within MEGA6 software. The scale bar represents a 2% nucleotide sequence divergence

**Figure 2:**
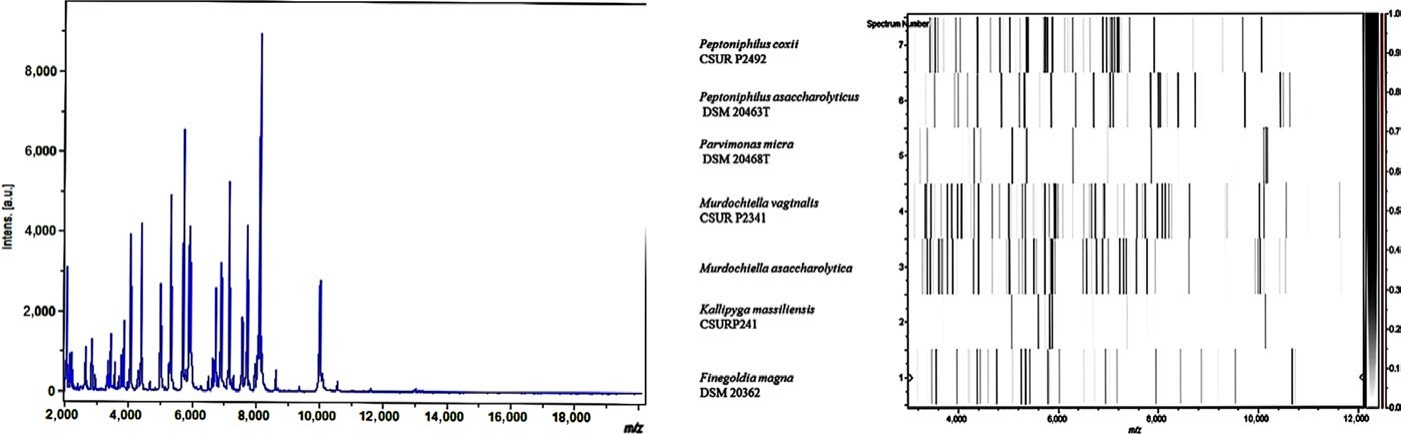
MALDI‐TOF information. (**a**) Reference mass spectrum from *Murdochiella vaginalis* strain Marseille‐P2341T spectra. (**b**) Gel view comparing *M. vaginalis* strain Marseille‐P2341T to other species within Peptoniphilaceae family. The gel view displays the raw spectra of loaded spectrum files arranged with a pseudo‐gel like appearance. The *x*‐axis records the *m*/*z* value. The left *y*‐axis displays the running spectrum number originating from subsequent spectra loading. The peak intensity is expressed by a gray scale scheme code. The right *y*‐axis indicates the relation between the color of a peak and its intensity, in arbitrary units. Displayed species are indicated on the left

Strain Marseille‐P2341^T^ was first isolated after 15 days of pre‐incubation of a vaginal sample in a blood culture bottle supplemented with rumen and sheep's blood under anaerobic conditions and then sub‐cultured on Schaedler agar. A score of 1.3 was also obtained with MALDI‐TOF MS identification, suggesting that this isolate was not in the database. The 16S rRNA gene sequence (accession number LT576397) of the strain exhibited 97.3% nucleotide sequence similarity with *M. asaccharolytica,* the phylogenetically‐closest species with a validly published name (Figure 1). As this value was lower than 98.7%, the threshold recommended for delineating a new species (Kim et al., 2014; Stackebrandt & Ebers, 2006), strain Marseille‐P2341^T^ was classified as a new species named *M. vaginalis* (Table 1). The reference spectrum of the strain Marseille‐P2341^T^ (Figure 2a) was then added to our database and compared to other known species of the family *Peptoniphilaceae* (Johnson, Whitehead, Cotta, Rhoades, & Lawson, 2014). Their differences are shown in the gel view which was obtained (Figure 2b).

**Table 1:** Classification and general features of *Murdochiella vaginalis* Marseille‐P2341^T^

Properties	Terms
Taxonomy	**Kingdom**:* Bacteria*
	**Phylum**:* Firmicutes*
	**Class**:* Clostridia*
	**Order**:* Clostridiales*
	**Family**:* Peptoniphiliaceae*
	**Genus**:* Murdochiella*
	**Species**:* M. vaginalis*
Type strain	Marseille‐P2341^T^
Isolation site	Human vagina
Isolation country	France
Gram stain	Positive
Cell shape	Coccus
Motility	No
Oxygen requirements	Anaerobic
Optimal temperature	37°C
Temperature range	Mesophilic

### 3.2 Phenotypic characteristics

**Figure 3:**
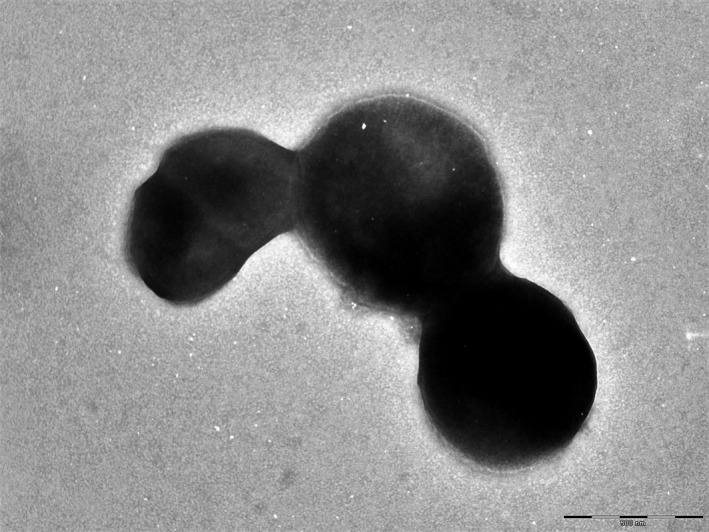
Transmission electron microscopy of *Murdochiella vaginalis* strain Marseille‐P2341^T^, using a Tecnai G20 transmission electron microscope (FEI Company). The scale bar represents 100 nm

Only grown in anaerobic conditions, strain Marseille‐P2341^T^ grows at temperatures between 25 to 42°C, with optimal growth at 37°C after 48 hr of incubation. It needs NaCl concentrations lower than 5 g/L and a pH ranging from 6.5 to 8.5. After 2 days of incubation at 37°C under anaerobic conditions on Columbia agar (bioMérieux), colonies are circular, white, and opaque with a diameter of 2–2.5 mm. Gram‐staining shows a Gram‐positive coccus. Individual cells show a diameter ranging from 0.6 to 0.8 μm under an electron microscope (Figure 3). Nonmotile and nonspore‐forming, strain Marseille‐P2341^T^ exhibited positive oxidase activity. Nevertheless, catalase activity was negative and nitrate was not reduced.

Using an API ZYM strip, positive reactions were observed for leucine arylamidase, Naphtol‐AS‐BI‐phosphohydrolase, α and β‐galactosidase, glucosidase (α and β), N‐acetyl‐β‐glucosaminidase, α‐mannosidase, and α‐fucosidase. Alkaline phosphatase, lipases, and other reactions were negative. On an API 20A strip, we observed an acidification of glucose and an API 50CH strip revealed that only galactose, glucose, mannose, and potassium 5‐ketogluconate were metabolized. All the other reactions were negative on both API strips. The most abundant fatty acids found were 9‐Octadecenoic acid and Hexadecanoic acid (28% and 24%, respectively). Interesting minor fatty acids (<1%) are also described (Table 2). Cells were susceptible to oxacillin, penicillin, ceftriaxone, ciprofloxacin, clindamycin, doxycycline, erythromycin, fosfomycin, gentamycin, trimethoprim‐sulfamethoxazole, rifampicin, and vancomycin but resistant to colistin. The phenotypic characteristics of strain Marseille‐P2341^T^ were compared to those of closely related species and are summarized in Table 3 (Collins, 2004; Ezaki et al., 2001; Ezaki, Yamamoto, Ninomiya, Suzuki, & Yabuuchi, 1983; Murdoch & Shah, 1999; Tindall & Euzeby, 2006; Ulger‐Toprak et al., 2010).

**Table 2:** Cellular fatty acid composition (%)

Fatty acids	Name	Mean relative % (a)
18:1n9	9‐Octadecenoic acid	27.7 ± 6.6
16:0	Hexadecanoic acid	24.2 ± 4.1
18:2n6	9,12‐Octadecadienoic acid	15.7 ± 4.4
18:0	Octadecanoic acid	13.4 ± 2.2
14:0	Tetradecanoic acid	5.9 ± 7.0
18:1n7	11‐Octadecenoic acid	3.7 ± 0.6
15:0 iso	13‐methyl‐tetradecanoic acid	1.4 ± 1.7
17:0	Heptadecanoic acid	1.0 ± 0.1
14:0 3‐OH	3‐hydroxy‐Tetradecanoic acid	TR
20:0	Eicosanoic acid	TR
18:0 9,10‐methylene	2‐octyl‐Cyclopropaneoctanoic acid	TR
5:0 iso	3‐methyl‐butanoic acid	TR
20:4n6	5,8,11,14‐Eicosatetraenoic acid	TR
15:0	Pentadecanoic acid	TR
16:1n5	11‐Hexadecenoic acid	TR
17:0 anteiso	14‐methyl‐Hexadecanoic acid	TR
17:0 iso	15‐methyl‐Hexadecanoic acid	TR
20:1n9	11‐Eicosenoic acid	TR
15:0 anteiso	12‐methyl‐tetradecanoic acid	TR
17:1n7	10‐Heptadecenoic acid	TR
10:0	Decanoic acid	TR
20:2n6	11,14‐Eicosadienoic acid	TR
12:0	Dodecanoic acid	TR
19:0	Nonadecanoic acid	TR
22:5n2	7,10,13,16,19‐docosapentaenoic acid	TR
16:0 9,10‐methylene	2‐Hexyl‐Cyclopropaneoctanoic acid	TR
13:0	Tridecanoic acid	TR
4:0	Butanoic acid	TR
22:6n3	4,7,10,13,16,19‐Docosahexaenoic acid	TR

Mean peak area percentage; TR = trace amounts <1%.

**Table 3:** Differential characteristics of *Murdochiella vaginalis* and the phylogenetically related species*. Murdochiella vaginalis* strain Marseille‐P2341^T^, *Murdochiella asaccharolytica* strain WAL 1855C^T^, *Finegoldia magna* strain CCUG 17636^T^, *Peptoniphilus indolicus* ATCC 29427^T^, *Parvimonas micra* CCUG 46357^T^, *Helcococcus sueciensis* CCUG 47334^T^, and *Anaerococcus hydrogenalis* JCM 7635^T^

Properties	*M. vaginalis*	*M. asaccharolytica*	*F. magna*	*P. indolicus*	*P. micra*	*H. sueciensis*	*A. hydrogenalis*
Cell diameter (μm)	0.6–0.8	0.5–0.6	0.8–1.6	0.7–1.6	0.3–0.7	na	0.7–1.8
Oxygen requirement	Anaerobic	Anaerobic	Anaerobic	Anaerobic	Anaerobic	Facultative anaerobic	Anaerobic
DNA G+C content (mol%)	49.5	na	na	31.69	28.65	29.5	29.64
Production of							
Alkaline phosphatase	−	−	Variable	+	+	+	−
Indole	−	−	−	+	−	−	+
Catalase	−	−	Variable	na	Variable	−	−
Nitrate reductase	−	−	−	+	−	−	−
Urease	−	−	−	−	−	−	Variable
β‐galactosidase	+	−	−	−	−	−	‐
N‐acetyl‐glucosamine	+	−	−	na	−	+	na
Acid from							
Mannose	+	−	−	−	−	−	+
Glucose	+	−	−	−	−	+	+
Lactose	−	−	−	−	−	+	+
Raffinose	−	−	−	−	−	−	+
Habitat	Vaginal discharges	Human wound	Human specimen	Summer mastitis of cattle	Human specimen	Human wound	Vaginal discharges

+, positive reaction; −, negative reaction; na, data not available.

### 3.3 Genome properties

**Figure 4:**
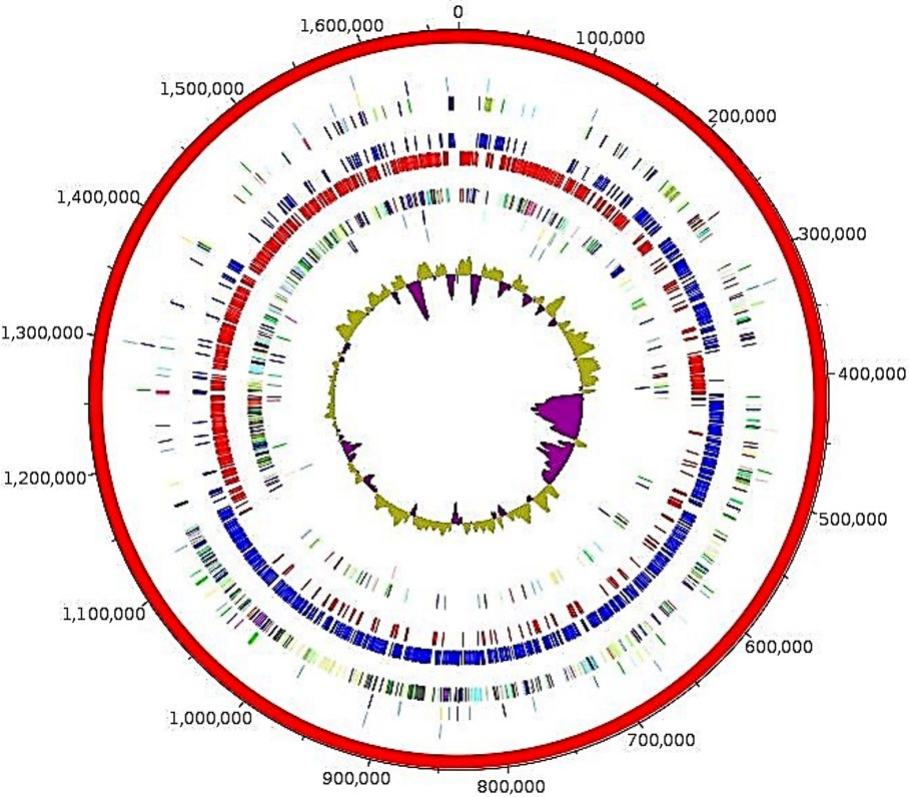
Graphical circular map of the genome. From outside to the center: Contigs (red/gray), COG category of genes on the forward strand (three circles), genes on forward strand (blue circle), genes on the reverse strand (red circle), COG category on the reverse strand (three circles), GC content

The genome measures 1,671,491 bp long and has 49.48 mol% of G+C content (Table 4, Figure 4). It is composed of one scaffold composed of one contig. Of the 1,501 predicted genes, 1,446 were protein‐coding genes and 55 were RNAs (two genes were 5S rRNA, two genes were 16S rRNA, two genes were 23S rRNA, 49 genes were tRNA genes). A total of 1,056 genes (73.03%) were assigned a putative function (by cogs or by NR blast). 56 genes were identified as ORFans (3.87%). The remaining 292 genes were annotated as hypothetical proteins (20.19%). Genome statistics are summarized in Table 4 and the distribution of the genes in COGs functional categories is presented in Table 5.

**Table 4:** Nucleotide content and gene count levels of the genome

Attribute	Value	% of total^a^
Size (bp)	1,671,491	100
G+C content (bp)	827,028	49.48
Coding region (bp)	1,511,436	90.42
Total genes	1,501	100
RNA genes	55	3.66
Protein‐coding genes	1,446	100
Genes with function prediction	1,056	73.03
Genes assigned to COGs	965	66.74
Genes with peptide signals	160	11.06
Genes with transmembrane helices	369	25.52

a The total is based on either the size of the genome in base pairs or the total number of protein coding genes in the annotated genome.

**Table 5:** Number of genes associated with the 25 general COG functional categories

Code	Value	% of total	Description
[J]	157	10.857538	Translation
[A]	0	0	RNA processing and modification
[K]	71	4.910097	Transcription
[L]	57	3.9419088	Replication, recombination and repair
[B]	0	0	Chromatin structure and dynamics
[D]	16	1.1065007	Cell cycle control, mitosis and meiosis
[Y]	0	0	Nuclear structure
[V]	45	3.1120331	Defense mechanisms
[T]	32	2.2130015	Signal transduction mechanisms
[M]	44	3.042877	Cell wall/membrane biogenesis
[N]	4	0.2766252	Cell motility
[Z]	0	0	Cytoskeleton
[W]	1	0.0691563	Extracellular structures
[U]	15	1.0373445	Intracellular trafficking and secretion
[O]	53	3.6652837	Post‐translational modification, protein turnover, chaperones
[X]	8	0.5532504	Mobilome: prophages, transposons
[C]	60	4.149378	Energy production and conversion
[G]	81	5.60166	Carbohydrate transport and metabolism
[E]	80	5.5325036	Amino acid transport and metabolism
[F]	51	3.526971	Nucleotide transport and metabolism
[H]	52	3.5961275	Coenzyme transport and metabolism
[I]	34	2.351314	Lipid transport and metabolism
[P]	46	3.1811898	Inorganic ion transport and metabolism
[Q]	9	0.62240666	Secondary metabolites biosynthesis, transport and catabolism
[R]	92	6.3623796	General function prediction only
[S]	42	2.9045644	Function unknown
_	481	33.26418	Not in COGs

### 3.4 Genomic comparison

**Figure 5:**
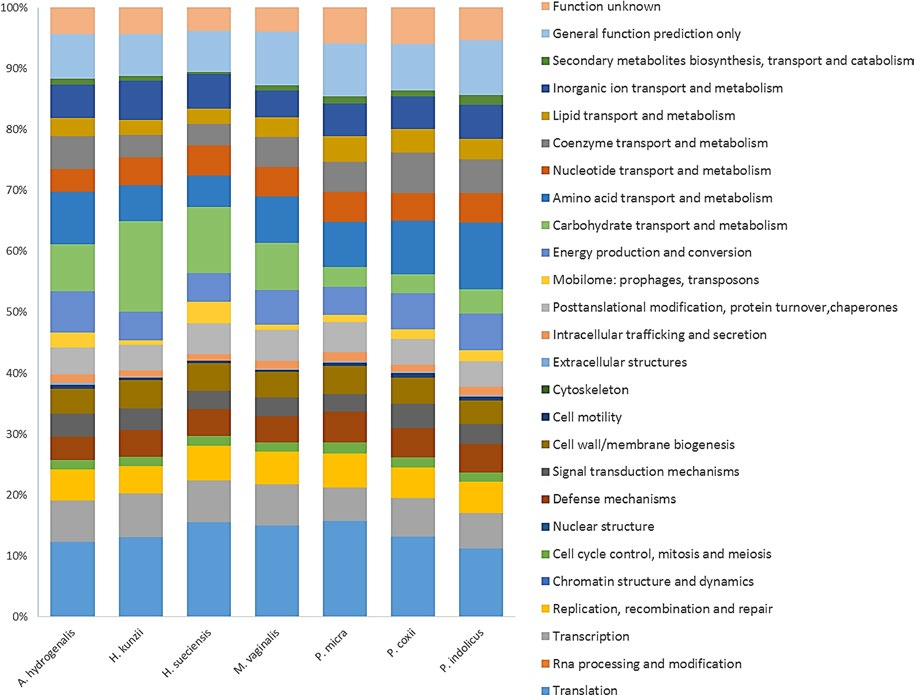
Distribution of functional classes of predicted genes according to the clusters of orthologous groups of proteins of *Murdochiella vaginalis* strain Marseille‐P2341T among other species

The comparison of the genome of our species with the closest related species (Table 6) reveals that the genome sequence of strain Marseille‐P2341^T^ (1.67 Mbp) is larger than that of *Helcococcus sueciensis* (1.57 Mbp), but smaller than those of *Parvimonas micra*,* Peptoniphilus coxii*,* Anaerococcus hydrogenalis*,* Helcococcus kunzii*, and *Peptoniphilus indolicus* (1.70, 1.84, 1.89, 2.10, and 2.24, respectively). The G+C content of strain Marseille‐P2341 ^T^ (49.48 mol%) is greater than those of all compared species. The gene content of strain Marseille‐P2341^T^ (1,446) is almost equal to that of *H. sueciensis* but is smaller than those of other compared genomes. However, in all the compared genomes, the distribution of genes in COG categories was similar. Nevertheless, there are fewer genes of *M. vaginalis* present in the COG categories X (Mobilome: prophages, transposons) and W (Extracellular structures) than other compared species (Figure 5). Moreover, the AGIOS analysis shows that strain Marseille‐P2341^T^ shares between 509 and 542 orthologous genes with closely related species (Table 7) and analysis of the average percentage of nucleotide sequence identity ranged from 50.8% to 56.4% with *P. micra* and *H. sueciensis*, respectively (Table 7). In addition, the digital DNA‐DNA hybridization (dDDH) of strain Marseille‐P2341^T^ and its closest species varied between 22.40% to 36% with 22.40, 23.60, 23.70, 25.50, 25.90, and 36% for *H. kunzii, A. hydrogenalis, P. micra, P. coxii, H. sueciensis*, and *P. indolicus,* respectively. Unfortunately, *M. asaccharolytica* was not included in this comparison because its genome was not sequenced.

**Table 6:** Genome comparison of closely related species to *Murdochiella vaginalis* strain Marseille‐P2341^T^

Species	INSDC identifier	Size (Mb)	G+C (mol%)	Gene Content
*M. vaginalis* strain Marseille‐P2341^T^	LT632322	1.671	49.48	1,501
*Anaerococcus hydrogenalis* DSM 7454	ABXA00000000.1	1.89	29.64	2,069
*Helcococcus kunzii* NCFB 2900	AGEI00000000.1	2.10	29.35	1,882
*Peptoniphilus indolicus* ATCC 29427	AGBB00000000.1	2.24	31.69	2,269
*Helcococcus sueciensis* CCUG 47334	AUHK00000000.1	1.57	28.40	1,445
*Peptoniphilus coxii* RMA 16757	LSDG00000000.1	1.84	44.62	1,86
*Parvimonas micra* ATCC 33270	ABEE00000000.2	1.70	28.65	1,678

INSDC, International Nucleotide Sequence Database Collaboration.

**Table 7:** Numbers of orthologous proteins shared between genomes (upper right) and AGIOS values obtained (lower left)

	*Murdochiella vaginalis*	*Anaerococcus hydrogenalis*	*Helcococcus kunzii*	*Parvimonas micra*	*Helcococcus sueciensis*	*Peptoniphilus indolicus*	*Peptoniphilus coxii*
*M. vaginalis*	**1,446**	538	514	511	509	525	542
*A. hydrogenalis*	51.39	**2,069**	538	516	526	565	580
*H. kunzii*	51.12	57.33	**1,882**	541	653	511	534
*P. micra*	50.80	57.96	59.47	**1,678**	530	533	534
*H. sueciensis*	56.37	59.46	63.43	58.83	**1,445**	491	514
*P. indolicus*	52.45	58.27	56.33	58.43	59.21	**2,269**	614
*P. coxii*	52.67	53.15	52.95	53.78	50.25	52.93	**1,860**

The numbers of proteins per genome are indicated in bold.

## 4 DISCUSSION

During the study of vaginal microbiota using culturomics, with the aim of exploring the vaginal flora as exhaustively as possible and identifying the bacteria involved in bacterial vaginosis in order to better manage this infection, strain Marseille‐P2341^T^ was identified in the vaginal sample of a patient suffering from bacterial vaginosis. Its phenotypic characteristics, MALDI‐TOF MS, 16S rRNA gene sequencing, and genome comparison with close phylogenic relatives enabled us to classify strain Marseille‐P2341^T^ as a new species of the genus *Murdochiella*. The 16S rRNA gene sequence similarity was 97.3% with *M. asaccharolytica*, which was lower than the 98.7% threshold recommended for defining a new species (Kim et al., 2014; Stackebrandt & Ebers, 2006). Created in 2010, the genus *Murdochiella* contains Gram‐positive staining anaerobic cocci bacteria which have been detected in human clinical samples (Ulger‐Toprak et al., 2010). Members of this genus are nonmotile and nonsporulating, as observed for strain Marseille‐P2341^T^.

A polyphasic taxono‐genomic strategy based on the combination of phenotypic and genomic analyses (Fournier & Drancourt, 2015; Fournier et al., 2015) was used to describe the new species whose strain Marseille‐P2341^T^ is the type strain. Strain Marseille‐P2341^T^ exhibited a specific MALDI‐TOF MS spectrum and differed from the other studied closed bacterial species in their fermentation of carbohydrate. Bacteria in the *Murdochiella* genus are asaccharolytic and do not ferment carbohydrates. However, the *M. vaginalis* strain Marseille‐P2341^T^ produces acid from glucose and mannose. This observation was confirmed by the annotation of the genome with the COGs database (Figure 5), showed that 7.7% of Marseille‐P2341 predicted genes' were dedicated to carbohydrate transport and metabolism functions. These genes include carbohydrate enzymes such as glucose‐6‐phosphate isomerase, 6‐phosphogluconolactonase, 6‐phosphofructokinase, fructose‐bisphosphate aldolase, triose‐phosphate isomerase, glyceraldehyde‐3‐phosphate dehydrogenase, 3‐phosphoglycerate kinase, phosphoglycerate mutase, enolase, pyruvate kinase, phosphomannomutase involved in carbohydrate metabolism, mainly in the process of glucose, fructose, and mannose metabolism.

The G+C content of strain Marseille‐P2341^T^ and its phylogenetically‐closest species ranges from 28.40 to 49.48 mol% and, as previously demonstrated, the difference in the G+C content is, at most, 1% in a species. Thus, overall, these values justify the strain Marseille‐P2341^T^ being classified as a distinct species. The AGIOS and GGDC values also confirm it belongs to a new species (Klenk, Meier‐Kolthoff, & Göker, 2014).

## 5 TAXONOMIC AND NOMENCLATURE PROPOSAL

### 5.1 Description of *Murdochiella vaginalis* sp. nov

*Murdochiella vaginalis* (va.gi.na'lis. L. n. *vagina*, sheath, vagina; L. fem. suff. *–alis,* suffix denoting pertaining to; N.L. fem. adj. *vaginalis*, pertaining to the vagina, of the vagina).

Obligate anaerobic *M. vaginalis* cells are Gram‐stain‐positive and coccus‐shaped. They are nearly 0.7 μm in diameter, nonmotile, nonspore‐forming, mesophilic, and occur in pairs or short chains. After 2 days of incubation on Columbia agar with 5% sheep's blood (bioMérieux) at 37°C under anaerobic conditions, colonies appear circular, white, and opaque with a diameter of 2–2.5 mm. Nitrate is not reduced; catalase and urease are also negative. Weakly saccharolytic, acid is produced only from glucose, mannose, and galactose. Positive reactions are observed for leucine arylamidase, Naphtol‐AS‐BI‐phosphohydrolase, α‐ galactosidase, β‐galactosidase, α‐glucosidase, β‐glucosidase, N‐acetyl‐β‐glucosaminidase, α‐mannosidase, and α‐fucosidase. The most abundant fatty acids are C_18:1n9_ (27.7%) and C_16:0_ (24.4%). The type strain is susceptible to oxacillin, penicillin, ceftriaxone, ciprofloxacin, clindamycin, doxycycline, erythromycin, fosfomycin, gentamycin, trimethoprim‐sulfamethoxazole, vancomycin, and rifampicin but resistant to colistin.

Its genome contains 49.48 mol% of G+C content and measures 1,671,491 bp long. The 16S rRNA and whole‐genome sequences are both deposited in EMBL‐EBI under accession numbers LT576397 and LT632322 respectively. The type strain Marseille‐P2341^T^ (=DSM 102237, =CSUR P2341) was isolated from the vaginal sample of a French woman suffering from bacterial vaginosis.

## CONFLICT OF INTEREST

The authors declare no conflict of interest.

## References

[mbo3570-bib-0001] Bohbot, J. M. , & Lepargneur, J. P. (2012). La vaginose en 2011: Encore beaucoup d'interrogations. Gynécologie Obstétrique & Fertilité, 40, 31–36. 10.1016/j.gyobfe.2011.10.013 22197267

[mbo3570-bib-0002] Bretelle, F. , Rozenberg, P. , Pascal, A. , Favre, R. , Bohec, C. , Loundou, A. , … Groupe de Recherche en Obstetrique Gynecologie (2015). High *Atopobium vaginae* and *Gardnerella vaginalis* vaginal loads are associated with preterm birth. Clinical Infectious Diseases, 60, 860–867. 10.1093/cid/ciu966 25452591

[mbo3570-bib-0003] Carver, T. , Harris, S. R. , Berriman, M. , Parkhill, J. , & McQuillan, J. A. (2012). Artemis: An integrated platform for visualization and analysis of high‐throughput sequence‐based experimental data. Bioinformatics, 28, 464–469. 10.1093/bioinformatics/btr703 22199388 PMC3278759

[mbo3570-bib-0004] Carver, T. , Thomson, N. , Bleasby, A. , Berriman, M. , & Parkhill, J. (2009). DNAPlotter: Circular and linear interactive genome visualization. Bioinformatics, 25, 119–120. 10.1093/bioinformatics/btn578 18990721 PMC2612626

[mbo3570-bib-0005] Chan, J. Z. , Halachev, M. R. , Loman, N. J. , Constantinidou, C. , & Pallen, M. J. (2012). Defining bacterial species in the genomic era: Insights from the genus *Acinetobacter* . BMC Microbiology, 12, 302 10.1186/1471-2180-12-302 23259572 PMC3556118

[mbo3570-bib-0006] Clark, K. , Karsch‐Mizrachi, I. , Lipman, D. J. , Ostell, J. , & Sayers, E. W. (2016). GenBank. Nucleic Acids Research, 44, D67–D72. 10.1093/nar/gkv1276 26590407 PMC4702903

[mbo3570-bib-0007] Collins, M. D. (2004). *Helcococcus sueciensis* sp. nov., isolated from a human wound. International Journal of Systematic and Evolutionary Microbiology, 54, 1557–1560. 10.1099/ijs.0.63077-0 15388710

[mbo3570-bib-0008] De Vos, P. , Garrity, G. M. , Jones, D. , Krieg, N. R. , Ludwig, W. , Rainey, F. A. , … Whitman, W. B. (2009). The Firmicutes In Garrity G. M. (Ed.), Bergey's Manual of Systematic Bacteriology (pp. 465–511). New York, NY: Springer.

[mbo3570-bib-0009] Dione, N. , Sankar, S. A. , Lagier, J. C. , Khelaifia, S. , Michele, C. , Armstrong, N. , … Fournier, P. E. (2016). Genome sequence and description of *Anaerosalibacter massiliensis* sp. nov. New Microbes and New Infections, 10, 66–76. 10.1016/j.nmni.2016.01.002 26937282 PMC4753391

[mbo3570-bib-0010] Drancourt, M. , Bollet, C. , Carlioz, A. , Martelin, R. , Gayral, J.‐P. , & Raoult, D. (2000). 16S ribosomal DNA sequence analysis of a large collection of environmental and clinical unidentifiable bacterial isolates. Journal of Clinical Microbiology, 38, 3623–3630. 11015374 10.1128/jcm.38.10.3623-3630.2000 PMC87447

[mbo3570-bib-0011] Ezaki, T. , Kawamura, Y. , Li, N. , Li, Z. Y. , Zhao, L. , & Shu, S. (2001). Proposal of the genera *Anaerococcus* gen. nov., *Peptoniphilus* gen. nov. and *Gallicola* gen. nov. for members of the genus Peptostreptococcus. International Journal of Systematic and Evolutionary Microbiology, 51, 1521–1528. 10.1099/00207713-51-4-1521 11491354

[mbo3570-bib-0012] Ezaki, T. , Yamamoto, N. , Ninomiya, K. , Suzuki, S. , & Yabuuchi, E. (1983). Transfer of *Peptococcus indolicus, Peptococcus asaccharolyticus, Peptococcus prevotii*, and *Peptococcus magnus* to the Genus *Peptostreptococcus* and Proposal of *Peptostreptococcus tetradius* sp. nov. International Journal of Systematic and Evolutionary Microbiology, 33, 683–698. 10.1099/00207713-33-4-683

[mbo3570-bib-0013] Fournier, P. E. , & Drancourt, M. (2015). New Microbes New Infections promotes modern prokaryotic taxonomy: A new section “TaxonoGenomics: New genomes of microorganisms in humans”. New Microbes and New Infections, 7, 48–49. 10.1016/j.nmni.2015.06.001 26199732 PMC4506979

[mbo3570-bib-0014] Fournier, P. E. , Lagier, J. C. , Dubourg, G. , & Raoult, D. (2015). From culturomics to taxonomogenomics: A need to change the taxonomy of prokaryotes in clinical microbiology. Anaerobe, 36, 73–78. 10.1016/j.anaerobe.2015.10.011 26514403

[mbo3570-bib-0015] Fredricks, D. N. , Fiedler, T. L. , & Marrazzo, J. M. (2005). Molecular identification of bacteria associated with bacterial vaginosis. New England Journal of Medicine, 353, 1899–1911. 10.1056/NEJMoa043802 16267321

[mbo3570-bib-0016] Gouret, P. , Paganini, J. , Dainat, J. , Louati, D. , Darbo, E. , Pontarotti, P. , & Levasseur, A. (2011). Integration of evolutionary biology concepts for functional annotation and automation of complex research in evolution: The multi‐agent software system DAGOBAH In Pontarotti P. (Ed.), Evolutionary biology – Concepts, biodiversity, macroevolution and genome evolution (pp. 71–87). New York, NY: Springer, Berlin Heidelberg 10.1007/978-3-642-20763-1

[mbo3570-bib-0017] Gouret, P. , Vitiello, V. , Balandraud, N. , Gilles, A. , Pontarotti, P. , & Danchin, E. G. (2005). FIGENIX: Intelligent automation of genomic annotation: Expertise integration in a new software platform. BMC Bioinformatics, 6, 198 10.1186/1471-2105-6-198 16083500 PMC1188056

[mbo3570-bib-0018] Hyatt, D. , Chen, G. L. , LoCascio, P. F. , Land, M. L. , Larimer, F. W. , & Hauser, L. J. (2010). Prodigal: Prokaryotic gene recognition and translation initiation site identification. BMC Bioinformatics, 11, 119 10.1186/1471-2105-11-119 20211023 PMC2848648

[mbo3570-bib-0019] Johnson, C. N. , Whitehead, T. R. , Cotta, M. A. , Rhoades, R. E. , & Lawson, P. A. (2014). *Peptoniphilus stercorisuis* sp. nov., isolated from a swine manure storage tank and description of *Peptoniphilaceae* fam. nov. International Journal of Systematic and Evolutionary Microbiology, 64, 3538–3545. 10.1099/ijs.0.058941-0 25056296

[mbo3570-bib-0020] Kim, M. , Oh, H. S. , Park, S. C. , & Chun, J. (2014). Towards a taxonomic coherence between average nucleotide identity and 16S rRNA gene sequence similarity for species demarcation of prokaryotes. International Journal of Systematic and Evolutionary Microbiology, 64, 346–351. 10.1099/ijs.0.059774-0 24505072

[mbo3570-bib-0021] Klenk, H. P. , Meier‐Kolthoff, J. P. , & Göker, M. (2014). Taxonomic use of DNA G+C content and DNA–DNA hybridization in the genomic age. International Journal of Systematic and Evolutionary Microbiology, 64, 352–356. 10.1099/ijs.0.056994-0 24505073

[mbo3570-bib-0022] Lagesen, K. , Hallin, P. , Rodland, E. A. , Staerfeldt, H.‐H. , Rognes, T. , & Ussery, D. W. (2007). RNAmmer: Consistent and rapid annotation of ribosomal RNA genes. Nucleic Acids Research, 35, 3100–3108. 10.1093/nar/gkm160 17452365 PMC1888812

[mbo3570-bib-0023] Lagier, J. C. , Armougom, F. , Million, M. , Hugon, P. , Pagnier, I. , Robert, C. , … Trape, J. F. (2012). Microbial culturomics: Paradigm shift in the human gut microbiome study. Clinical Microbiology & Infection, 18, 1185–1193. 10.1111/1469-0691.12023 23033984

[mbo3570-bib-0024] Lagier, J. C. , Hugon, P. , Khelaifia, S. , Fournier, P. E. , La Scola, B. , & Raoult, D. (2015). The rebirth of culture in microbiology through the example of culturomics to study human gut microbiota. Clinical Microbiology Reviews, 28, 237–264. 10.1128/CMR.00014-14 25567229 PMC4284300

[mbo3570-bib-0025] Lamont, R. , Sobel, J. , Akins, R. , Hassan, S. , Chaiworapongsa, T. , Kusanovic, J. , & Romero, R. (2011). The vaginal microbiome: New information about genital tract flora using molecular based techniques. BJOG: An International Journal of Obstetrics and Gynaecology, 118, 533–549. 10.1111/j.1471-0528.2010.02840.x 21251190 PMC3055920

[mbo3570-bib-0026] Le Page, S. , van Belkum, A. , Fulchiron, C. , Huguet, R. , Raoult, D. , & Rolain, J.‐M. (2015). Evaluation of the PREVI^®^ Isola automated seeder system compared to reference manual inoculation for antibiotic susceptibility testing by the disk diffusion method. European Journal of Clinical Microbiology and Infectious Diseases, 34, 1859–1869. 10.1007/s10096-015-2424-8 26092031

[mbo3570-bib-0027] Lechner, M. , Findeiss, S. , Steiner, L. , Marz, M. , Stadler, P. F. , & Prohaska, S. J. (2011). Proteinortho: Detection of (Co‐) orthologs in large‐scale analysis. BMC Bioinformatics, 12, 124 10.1186/1471-2105-12-124 21526987 PMC3114741

[mbo3570-bib-0028] Lepargneur, J. P. , & Rousseau, V. (2002). Protective role of the Doderleïn flora. Journal de Gynecologie, Obstetrique et Biologie de la Reproduction, 31, 485–494. 12379833

[mbo3570-bib-0029] Lowe, T. M. , & Eddy, S. R. (1997). tRNAscan‐SE: A program for improved detection of transfer RNA genes in genomic sequence. Nucleic Acids Research, 25, 955–964. 10.1093/nar/25.5.0955 9023104 PMC146525

[mbo3570-bib-0030] Meier‐Kolthoff, J. P. , Auch, A. F. , Klenk, H. P. , & Göker, M. (2013). Genome sequence‐based species delimitation with confidence intervals and improved distance functions. BMC Bioinformatics, 14, 60 10.1186/1471-2105-14-60 23432962 PMC3665452

[mbo3570-bib-0031] Menard, J. , Fenollar, F. , Henry, M. , Bretelle, F. , & Raoult, D. (2008). Molecular Quantification of *Gardnerella vaginalis* and *Atopobium vaginae* Loads to Predict Bacterial Vaginosis. Clinical Infectious Diseases, 47, 33–43. 10.1086/588661 18513147

[mbo3570-bib-0032] Murdoch, D. , & Shah, H. N. (1999). Reclassification of *Peptostreptococcus magnus* (Prevot 1933) Holdeman and Moore 1972 as *Finegoldia magna* comb. nov. and Peptostreptococcus micros (Prevot 1933) Smith 1957 as *Micromonas micros* comb. nov. Anaerobe, 5, 555–559. 10.1006/anae.1999.0197

[mbo3570-bib-0033] Murray, P. R. , Baron, E. J. , Jorgensen, J. H. , Landry, M. L. , & Pfaller, M. A. (2007). Manual of clinical microbiology (9th ed.). Washington, D.C.: ASM Press.

[mbo3570-bib-0034] Pal, K. , Roy, S. , Behera, B. , Kumar, N. , Sagiri, S. , & Ray, S. (2011). Bacterial vaginosis: Etiology and modalities of treatment‐A brief note. Journal of Pharmacy And Bioallied Sciences, 3, 496 10.4103/0975-7406.90102 22219582 PMC3249696

[mbo3570-bib-0035] Pépin, J. , Deslandes, S. , Giroux, G. , Sobéla, F. , Khonde, N. , Diakité, S. , … Frost, E. (2011). The complex vaginal flora of west african women with bacterial vaginosis. PLoS ONE, 6, e25082 10.1371/journal.pone.0025082 21949860 PMC3176826

[mbo3570-bib-0036] Ramasamy, D. , Mishra, A. K. , Lagier, J. C. , Padhmanabhan, R. , Rossi, M. , Sentausa, E. , … Fournier, P. E. (2014). A polyphasic strategy incorporating genomic data for the taxonomic description of novel bacterial species. International Journal of Systematic and Evolutionary Microbiology, 64, 384–391. 10.1099/ijs.0.057091-0 24505076

[mbo3570-bib-0037] Sasser, M. (2006). Bacterial identification by gas chromatographic analysis of fatty acids methyl esters (GC‐FAME). MIDI, Technical Note.

[mbo3570-bib-0038] Seng, P. , Drancourt, M. , Gouriet, F. , La Scola, B. , Fournier, P. , Rolain, J. M. , & Raoult, D. (2009). Ongoing revolution in bacteriology: Routine identification of bacteria by matrix‐assisted laser desorption ionization time‐of‐flight mass spectrometry. Clinical Infectious Diseases, 49, 543–551. 10.1086/600885 19583519

[mbo3570-bib-0039] Shipitsyna, E. , Roos, A. , Datcu, R. , Hallén, A. , Fredlund, H. , Jensen, J. S. , … Unemo, M. (2013). Composition of the vaginal microbiota in women of reproductive age – Sensitive and specific molecular diagnosis of bacterial vaginosis is possible? PLoS ONE, 8, e60670 10.1371/journal.pone.0060670 23585843 PMC3621988

[mbo3570-bib-0040] Stackebrandt, E. , & Ebers, J. (2006). Taxonomic parameters revisited: Tarnished gold standards. Microbiology Today, 33, 152.

[mbo3570-bib-0041] Svare, J. , Schmidt, H. , Hansen, B. , & Lose, G. (2006). Bacterial vaginosis in a cohort of Danish pregnant women: Prevalence and relationship with preterm delivery, low birthweight and perinatal infections. BJOG: An International Journal of Obstetrics and Gynaecology, 113, 1419–1425. 10.1111/j.1471-0528.2006.01087.x 17010117

[mbo3570-bib-0042] Tindall, B. J. , & Euzeby, J. P. (2006). Proposal of *Parvimonas* gen. nov. and *Quatrionicoccus* gen. nov. as replacements for the illegitimate, prokaryotic, generic names *Micromonas* Murdoch and Shah 2000 and *Quadricoccus* Maszenan et al. 2002, respectively. International Journal of Systematic and Evolutionary Microbiology, 56, 2711–2713. 10.1099/ijs.0.64338-0 17082417

[mbo3570-bib-0043] Turovskiy, Y. , Sutyak Noll, K. , & Chikindas, M. L. (2011). The etiology of bacterial vaginosis. Journal of Applied Microbiology, 110, 1105–1128. 10.1111/j.1365-2672.2011.04977.x 21332897 PMC3072448

[mbo3570-bib-0044] Ulger‐Toprak, N. , Liu, C. , Summanen, P. H. , & Finegold, S. M. (2010). *Murdochiella asaccharolytica* gen. nov., sp. nov., a Gram‐stain‐positive, anaerobic coccus isolated from human wound specimens. International Journal of Systematic and Evolutionary Microbiology, 60, 1013–1016. 10.1099/ijs.0.015909-0 19666803

[mbo3570-bib-0045] Vandamme, P. , Pot, B. , Gillis, M. , De Vos, P. , Kersters, K. , & Swings, J. (1996). Polyphasic taxonomy, a consensus approach to bacterial systematics. Microbiological Reviews, 60, 407–438. 8801440 10.1128/mr.60.2.407-438.1996 PMC239450

