# EXPRESSION OF CONCERN: Characterization of a Novel Gram‐Stain‐Positive Anaerobic Coccus Isolated from the Female Genital Tract: Genome Sequence and Description of *Murdochiella vaginalis* sp. nov

**DOI:** 10.1002/mbo3.70245

**Published:** 2026-02-19

## Abstract

**EXPRESSION OF CONCERN**: K. Diop, A. Diop, S. Khelaifia, et al., *“*Characterization of a Novel Gram‐Stain‐Positive Anaerobic Coccus Isolated from the Female Genital Tract: Genome Sequence and Description of *Murdochiella vaginalis* sp. nov.,” *Microbiology Open* 7, no. 3 (2018): e00570, https://doi.org/10.1002/mbo3.570.

This Expression of Concern is for the above article, published online on 21 June 2018 in Wiley Online Library (https://wileyonlinelibrary.com), and has been issued by John Wiley & Sons Ltd. The Expression of Concern has been agreed due to questions raised about the study's adherence to French legal and ethical requirements for research involving human subjects. The investigation into these concerns is ongoing. Therefore, the journal has decided to issue an Expression of Concern to inform and alert readers.

